# Novel molecular markers for *Taxodium* breeding from the chloroplast genomes of four artificial *Taxodium* hybrids

**DOI:** 10.3389/fgene.2023.1193023

**Published:** 2023-08-02

**Authors:** Minghua Yue, Hong Chen, Lei Xuan, Ying Yang, Xinran Chong, Mingzhi Li, ChaoGuang Yu, Xiaoqing Lu, Fan Zhang

**Affiliations:** ^1^ Institute of Botany, Jiangsu Province and Chinese Academy of Sciences, Nanjing, China; ^2^ Nanjing Botanical Garden Mem. Sun Yat-Sen, Nanjing, China; ^3^ Jiangsu Key Laboratory for the Research and Utilization of Plant Resources, Nanjing, China; ^4^ Guangzhou Bio&Data Technology Co., Ltd., Guangzhou, China

**Keywords:** *Taxodium*, chloroplast genome, phylogenetic relationships, molecular markers, breeding

## Abstract

*Taxodium* “Zhongshanshan” are a group of intraspecific *Taxodium* hybrids with superparental dominance and high ecological and economic value in southern China. Identifying the parentage of hybrids, especially the male parent, is critically important for genetic studies. However, the large nuclear genomes of members of the genus *Taxodium* pose a major challenge for the development of molecular markers. Here, we developed novel molecular markers by conducting a comparative analysis of the chloroplast genomes of four artificial *Taxodium* hybrids and their parents. The lengths of the whole chloroplast genome ranged from 131,942 to 132,128 bp, and the total guanine (GC) content of the chloroplast genomes ranged from 34.6% to 35.81%. A total of 120 unique genes were identified, including 83 protein-coding genes, 33 transfer RNAs, and four ribosomal RNAs. There were 69‐71 simple sequence repeats were detected in the four hybrids. Phylogenetic analysis revealed that these hybrids clustered with their paternal parents. Similar findings were obtained by analysis of the GC content of protein-coding genes. Molecular markers were developed using the highly variable regions of the chloroplast genomes, and polymerase chain reaction (PCR) assays revealed that these markers were effective for identifying the male parents of these hybrids. Our findings indicate for the first time that the chloroplast genomes of *Taxodium* are paternally inherited. Generally, these molecular markers could facilitate breeding and genetic studies of *Taxodium*.

## 1 Introduction

The three members of the genus *Taxodium*, *Taxodium distichum* (L.) Rich (bald cypress), *T. mucronatum* Tenore (Montezuma cypress), and *T. ascendens* Brongn (pond cypress), are 30–40 m tall trees native to North America (Denny and Arnold, 2007). *T. distichum* is the most tolerant of flooding and strong winds; *T. mucronatum* is the most tolerant of saline and alkaline soils but is the most susceptible to red blight disease (Zhang et al., 2021); and *T. ascendens* is the most resistant to red blight disease but the least tolerant of flooding. All three of these species have been introduced to southeastern China for their ornamental and ecological value (Wang et al., 2017).

Interspecific hybridization can result in the production of new species; additionally, it can be used to generate hybrids with desirable characteristics of species crossed. Previously, we artificially hybridized the three *Taxodium* species to generate several new varieties, which were referred to as “Zhongshanshan”; these were selected from the progeny because of their superior features (Creech et al., 2011). For example, the desirable characteristics of *T*. “Zhongshanshan 302” (*T. distichum* × *T. mucronatum*) and *T*. “Zhongshanshan 401” (*T. ascendens* × *T. mucronatum*) included their high growth rate, ability to grow well in alkaline and saline soils, and high ornamental value (Wang et al., 2016). The growth rate of *T*. “Zhongshanshan 118” (*T*. “Zhongshanshan 302” ×*T. mucronatum*) is higher than that of its parents; the salt, alkali, and flood resistance of *T.* “Zhongshanshan 118” is also higher than that of its parents. Because of the desirable characteristics of these varieties, they have become increasingly popular in 18 provinces and municipalities in China (Xuan et al., 2021). Previously, expressed sequence tag-simple sequence repeat markers identified via transcriptome sequencing, genetic linkage maps built using sequence-related amplified polymorphism markers (Cheng et al., 2015), simple sequence repeat (SSR) markers (Wang et al., 2016), and high-density genetic maps built using specific locus amplified fragment sequencing technology (Yang et al., 2018) have greatly aided *Taxodium* breeding. Comprehensive genomic datasets of the genus *Taxodium* are needed to facilitate genetic studies and breeding. However, the large size of the nuclear genomes of members of the genus *Taxodium* makes generating these datasets a major challenge.

The chloroplast (cp) is a critically important organelle in green plants; it contains the photosynthetic machinery that plays a key role in carbon fixation, as well as plant growth and development (Palmer, 1987). The rate of evolutionary change of cp genomes is slower than that of nuclear genomes; genes and genome organization are more highly conserved in cp genomes than in nuclear genomes (Androsiuk et al., 2020). In addition, cp genomes are uniparentally inherited. Cp genomes have become increasingly used in phylogenetic analyses, as well as studies aimed at clarifying complex evolutionary relationships. Cp genome sequences have also been used for the development of molecular markers that can be used to distinguish morphologically similar species.

Here, we characterized the complete cp genomes of four *T*. “Zhongshanshan” varieties and compared them with the cp genome sequences of their parents. Phylogenetic analysis and analysis of the GC content of protein-coding genes revealed that these hybrids were most closely related to their male parents. Analysis of hypervariable regions was used to generate molecular markers that could be used to aid *Taxodium* breeding.

## 2 Materials and methods

### 2.1 Plant material and chloroplast genome sequencing, assembly, and annotation

The superior clones of *T*. “Zhongshanshan 302” (*T. distichum* × *T. mucronatum*), *T*. “Zhongshanshan 118” (*T*. “Zhongshanshan 302” × *T. mucronatum*), *T*. “Zhongshanshan 61” (*T. mucronatum* × *T. ascendens*), and *T*. “Zhongshanshan 401” (*T. ascendens* × *T. mucronatum*) were grown in a nursery at the Nanjing Botanical Garden (35°50′ N, 45°70′ E), Jiangsu Province, China. Fresh leaves of these plant were collected for DNA extraction with a modified CTAB method (Doyle and Doyle, 1987). DNA quality was assessed in a NanoDrop spectrophotometer (Thermo Scientific, Waltham, MA, United States), and its integrity and concentration were evaluated using 1% gel electrophoresis and a Qubit fluorometer (Life Technologies, Darmstadt, Germany), respectively.

The DNA library was sequenced by BGISEQ-500 platform (Bio&Data Biotechnologies Co. Ltd., Nanjing, China) with 150 based on sequencing by synthesis. Approximately 43 Mb of high-quality, clean paired-end reads was generated, and the sequences with cp-like reads were assembled with NOVOPlasty Version 4.3.1 (Dierckxsens et al., 2017).

The genome was annotated using GeSeq software (Tillich et al., 2017) and BLAST, and tRNAs were identified by tRNA scan-SE (Schattner et al., 2005). The results were further checked manually, and the circular graphical maps of the entire genome were drawn using the OGDRAW v1.3.1 program (http://ogdraw.mpimp-golm.mpg.de) (Lohse et al., 2007). The cp genome sequences of *T*. “Zhongshanshan” were deposited in the GenBank of NCBI (https://www.ncbi.nlm.nih.gov/) database under the accession numbers MW307789, MW307790, MW307791, and MW307792. The associated bioproject numbers are SRR13347061, SRR13347063, and SRR13347064.

### 2.2 Polymerase chain reaction assays

The highly variable regions were amplified with the high-fidelity polymerase of KOD-PlusNeo (TaKaRa, Dalian, China). The PCR reactions were performed in a total volume of 50 µL with 5 µL 10 × KOD-plus Neo buffer, 5 µL dNTP (2 mM), 3 µL MgSO_4_ (25 mM), 1.5 µL per primers (10 µM), 1 µL KOD-Plus Neo enzyme, and 100 ng genomic DNA. Thermal cycling consisted of 94°C for 2 min followed by 35 cycles of 98°C for 20 s, 62°C for 30 s, and 72 °C for 30 s and a final extension at 72 °C for 7 min 2.5% agarose gels were used to visualize the PCR products after electrophoresis.

### 2.3 Repeat sequence analysis and codon usage analysis

Simple sequence repeats (SSRs) were identified by MISA v2.1 (https://webblast.ipk-gatersleben.de/misa) software (Mudunuri and Nagarajaram, 2007) with the following parameters of mono-, di-, tri-, tetra-, pena-, and hexanucleotide being set as a minimum number of repeats of 10, 5, 4, 3, 3, and 3, respectively. The long repeat sequences, which included forward, reverse, palindrome, and complement repeats were analyzed by the REPuter (https://bibiserv.Cebitec.uni-bielefeld.de/reputer) software with the best top 50 sequences (Kurtz et al., 2001). The CodonW1.4.2 program was used to analyze the RSCU base on the protein-coding genes.

### 2.4 Chloroplast genome comparison

The mVISTA (http://genome.lbl.gov/vista/mvista) (Mayor et al., 2000) program was used to examine the genetic divergence among eight complete cp genomes, consisting of *T. distichum* (NC_034941), *T. distichum* (MN535013), *T*. “Zhongshanshan 61” (MW307790), *T. ascendens* (MN535012), *T*. “Zhongshanshan 118” (MW307791), *T*. “Zhongshanshan 302” (MW307792), *T*. “Zhongshanshan 401” (MW307789), and *T. mucronatum* (MN535011), in the Shuffle-LAGAN mode. BLAST Atlas on the GView server was used to assess the chloroplast genomes similarity with *T. distichum* (MN535013) genome as a reference (Petkau et al., 2010). DNaSP v5.0 software was used to calculate nucleotide diversity (Pi) among the eight *Taxodium* cp genomes. Pi values were calculated in 100 bp sliding windows with 25 bp steps. EMBOSS software was used to calculate the GC content at the first, second, and third codon positions (GC1, GC2, and GC3, respectively) and overall GC content of the genomes (Rice et al., 2000).

### 2.5 Phylogenetic analysis

For phylogenetic analysis, the whole cp genomes of eight *Taxodium* and an outgroup (*Cryptomeria japonica*) were downloaded from the National Center for Biotechnology Information (NCBI) database. MAFFT software (v7.450) was used to multiply sequence alignment of these cp genome sequences (Katoh et al., 2019). The maximum likelihood (ML) method based complete cp genome in IQ-TREE v2.1.4 was conducted for phylogenetic analysis with default parameter settings and 1,000 bootstrap replicates (Minh et al., 2020).

## 3 Results and discussion

### 3.1 Features of the *T*. “Zhongshanshan” cp genome

A total of 5.17–6.52 Gb of clean data of complete cp genomes were obtained from the four *T*. “Zhongshanshan” hybrids using the BGISEQ-500 platform (Supplementary Table S1). The cp genomes were *de novo* assembled using NOVOPlasty, and the length of the whole cp genome ranged from 131,942 bp for *T*. “Zhongshanshan 118” to 132,128 bp for *T*. “Zhongshanshan 61” (Supplementary Table S2).

**FIGURE 1:**
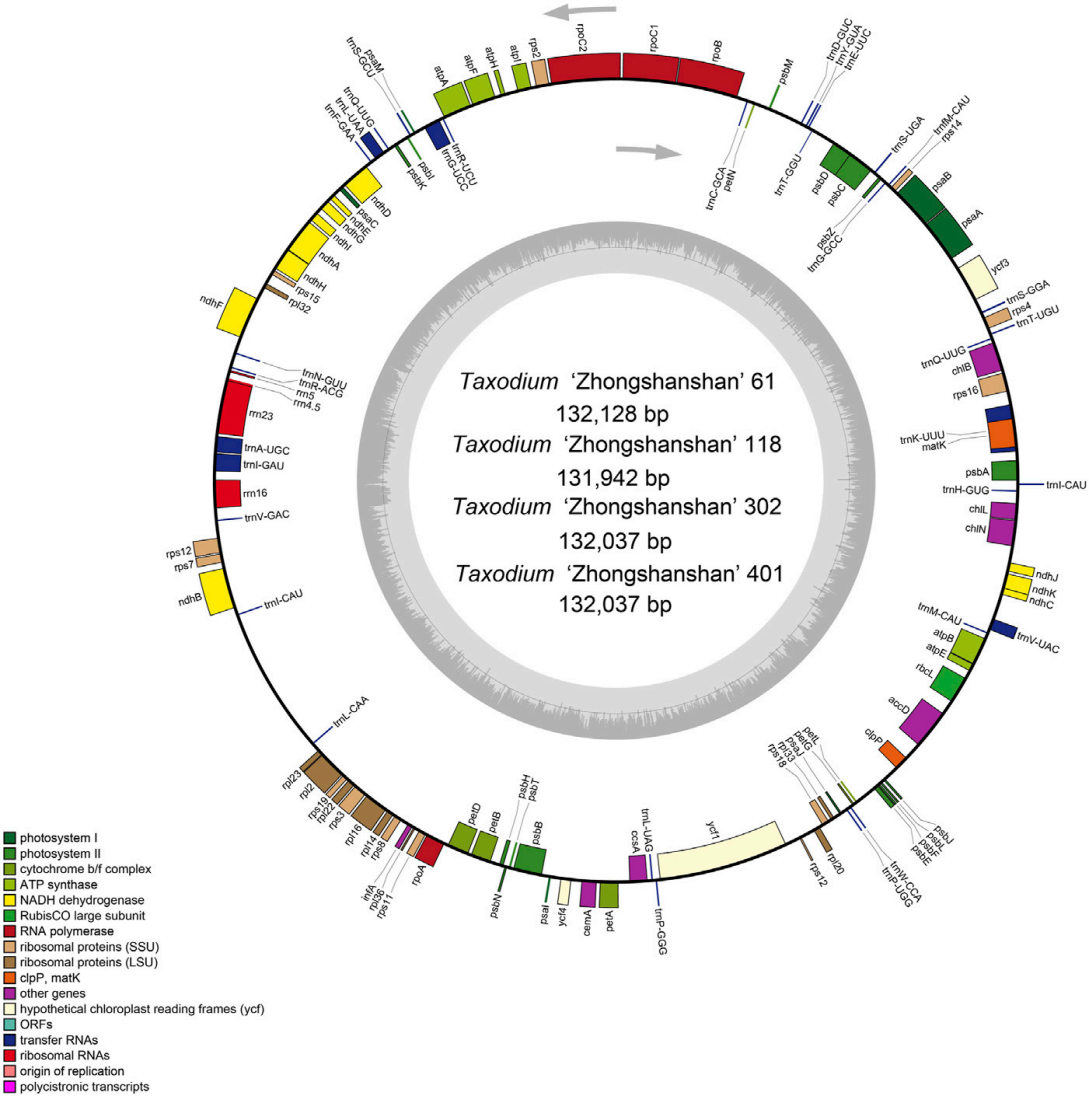
Map of the cp genome of *T*. “Zhongshanshan”. Genes drawn inside and outside of the circle are transcribed in a clockwise and a counter-clockwise direction, respectively. Different functional gene groups are color-coded. GC and AT contents are represented on the inner circle by dark and light gray, respectively.

The cp genome contains four characteristic regions: a large single-copy region (LSC) and a small single-copy region (SSC), which is separated by two inverted repeat (IRs) regions. Previous studies have shown that IRs play key roles determining the stability of the genome and the conservation of genes (Ping et al., 2022). However, the IR region is lacking in some plant families such as Pinaceae and Taxaceae (Yi et al., 2013). IR regions were absent in the cp genomes of the four hybrids (Figure 1), which is consistent with analyses of the cp genomes of their parents (Duan et al., 2020).

All four *T*. “Zhongshanshan” cp genomes contained 120 genes, including 83 protein-coding genes, 33 tRNAs, and four rRNAs; the distribution of these genes was consistent among all four “Zhongshansha” cp genomes examined. A total of 62 genes belonged to the self-replication category; 33 of these encoded tRNAs, 12 of these encoded small ribosomal subunit proteins, and nine of these encoded large ribosomal subunit proteins. Fifty of these genes were related to photosynthesis, including six genes that encoded ATP synthase subunits, 11 genes that encoded NADH-plastoquinone oxidoreductase subunits, six genes that encoded cytochrome b/f complex subunits, eight genes that encoded photosystem I subunits, 15 genes that encoded and PSII subunits, three genes that encoded photochlorophyllide reductase subunits, and one gene that encoded the large subunit of Rubisco. A total of six groups were in the biosynthesis category, and each group contained one gene (Table 1). A total of 17 intron-containing genes were identified in the *T*. “Zhongshanshan” cp genome; 15 of these genes contained two exons, and two of these genes (*ycf3* and *rps12*) contained three exons. Two copies of the *trnQ-UUG* and *trnI-CAU* genes were detected in the cp genomes of *T*. “Zhongshanshan” (Table 1). The AT content of the cp genome was 64.7%, which indicates that the nucleotide composition was AT-biased.

**TABLE 1:** List of genes in the cp genomes of four *T*. ‘Zhongshanshan’ in this study.

Category	Gene group	Name of gene	Number
Self-replication	Proteins of the large ribosomal subunit	*rpl2* ^a^, *rpl14*, *rpl16* ^a^, *rpl20*, *rpl22*, *rpl23*, *rpl32*, *rpl33*, *rpl36*	9
	Proteins of the small ribosomal subunit	*rps2*, *rps3*, *rps4*, *rps7, rps8*, *rps11*, *rps12* ^b^, *rps14*, *rps15, rps16* ^a^, *rps18*, *rps19*	12
	Subunits of RNA polymerase	*rpoA*, *rpoB*, *rpoC1* ^a^, *rpoC2*	4
	rRNAs	*rrn23S*, *rrn16S*, *rrn5S*, *rrn4.5S*	4
	tRNAs	*trnH-GUG*, *trnK-UUU* ^a^, *trnQ-UUG* ^c^, *trnS-GCU*, *trnG-UCC, trnR-UCU*, *trnC-GCA*, *trnD-GUC*, *trnY-GUA*, *trnE-UUC*, *trnT-GGU*, *trnS-UGA*, *trnfM-CAU*, *trnS-GGA*, *trnT-UGU*, *trnL-UAA* ^a^ *, trnF-GAA*, *trnV-UAC* ^a^, *trnM-CAU*, *trnW-CCA*, *trnP-UGG*, *trnI-CAU* ^c^, *trnV-GAC*, *trnI-GAU* ^a^, trnL-CAA, *trnA-UGC* ^a^, *trnR-ACG*, *trnN-GUU*, *trnL-UAG*, *trnP-GGG*, *trnG-GCC*	33
Photosynthesis	Subunits of photosystem I	*psaA*, *psaB*, *psaC*, *psaM*, *psaI*, *psaJ*, *ycf3* ^b^ *, ycf4*	8
	Submits of photosystem II	*psbA*, *psbB*, *psbC*, *psbD*, *psbE*, *psbF*, *psbH*, *psbI*, *psbJ*, *psbK*, *psbL*, *psbM*, *psbN*, *psbT*, *psbZ*	15
	Subunits of NADH NADH-plastoquinone oxidoreductase	*ndhA* ^a^, *ndhB* ^a^, *ndhC*, *ndhD*, *ndhE*, *ndhF*, *ndhG*, *ndhH*, *ndhI*, *ndhJ*, *ndhK*	11
	Subunits of cytochrome b/f complex	*petA*, *petB* ^a^, *petD* ^a^, *petG*, *petL*, *petN*	6
	Subunits of ATP synthase	*atpA*, *atpB*, *atpE*, *atpF* ^a^, *atpH*, *atpI*	6
	Large subunit of Rubisco	*rbcL*	1
	Subunits of photochlorophyllide reductase	*chlB*, *chlL*, *chlN*	3
Biosynthesis	Maturase	*matK*	1
	Protease	*clpP*	1
	Envelope membrane protein	*cemA*	1
	Acetyl-CoA carboxylase	*accD*	1
	c-type cytochrome synthesis gene	*ccsA*	1
	Translation initiation factor	*infA*	1
Unknown function	Conserved hypothetical chloroplast reading frames	*ycf1*, *ycf2*	2

^a^ Genes containing two exons; b. Genes containing three exons; c. Two copies.

### 3.2 Analysis of SSRs and long repeats

**FIGURE 2:**
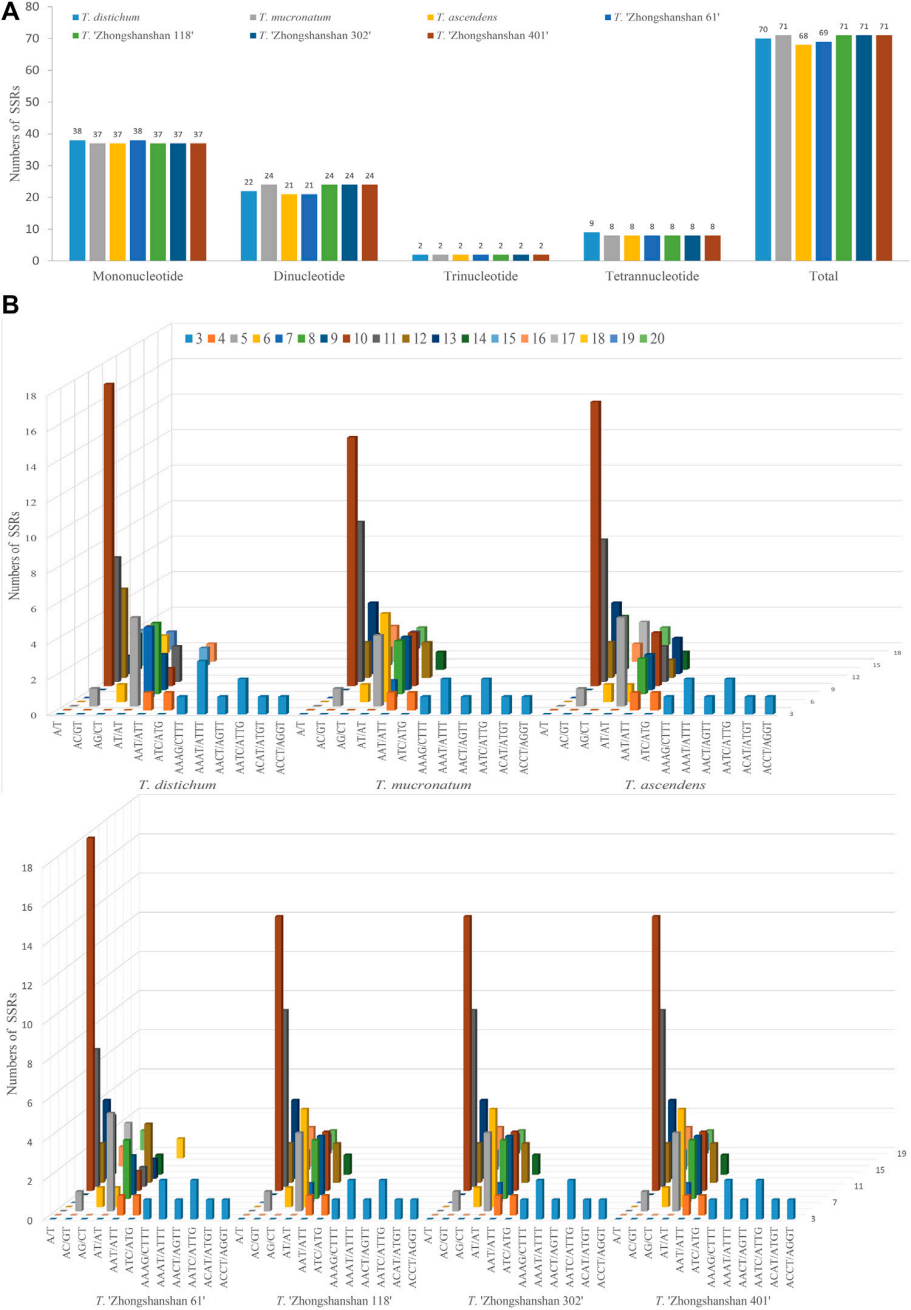
Analysis of SSRs in the cp genomes of eight *Taxodium* species. Number of different SSRs types **(A)** and SSR motifs **(B)** in the cp genomes of *Taxodium*.

To clarify differences between hybrid and parental cp genomes, a comparative analysis of the cp genomes of four new *T*. “Zhongshanshan” hybrids and their parents (*Taxodium distichum*, *T. mucronatum*, and *T. ascendens*) was conducted. 68–71 SSRs were detected in the cp genomes (Figure 2A). The most common motifs were mononucleotide SSRs (37–38, 52.11%–55.07%); mononucleotide A was the most common, followed by mononucleotide T (Supplementary Table S3). The number of trinucleotide and tetranucleotide repeats was consistent among the seven *Taxodium* cp genomes, with the exception of the cp genome of *T*. *distichum*, which had nine tetranucleotide repeats. The AT/AT sequence of dinucleotide SSRs was the most common (19–22, 27.53%–31.99%), followed by AC/GT (1, 0.01%) and AG/CT (1, 0.01%) (Figures 2B,C).

**FIGURE 3:**
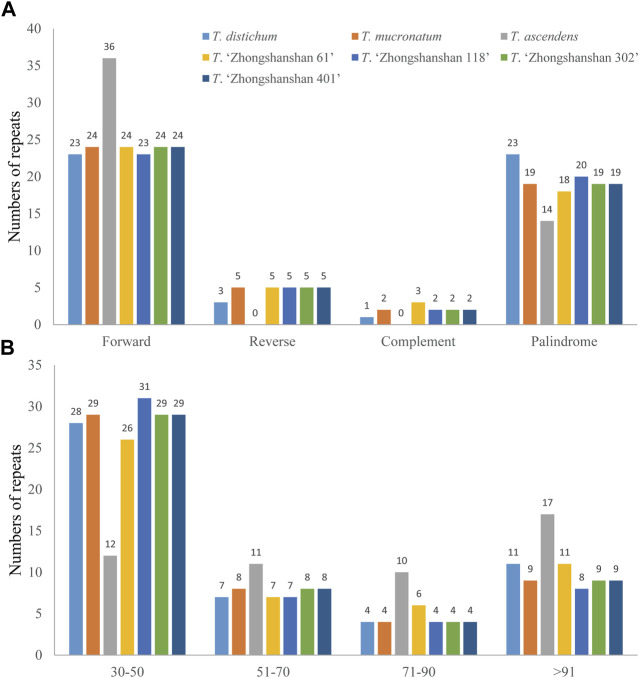
Comparison of long repeats in the cp genomes of seven Taxodium species. **(A)** Number of different types of long repeats. **(B)** Number of repeats in each repeat-length type.

Four types of long repeats (forward, palindromic, reverse, and complementary) were identified in the cp genomes of the seven *Taxodium* species examined. Forward repeats were the most common type in the cp genomes, followed by palindromic repeats (Figure 3A). Reverse and complementary repeats were absent in the cp genomes of *T. ascendens*, and five reverse repeats (with the exception of *T*. *distichum*, which has three reverse repeats) and two complementary repeats (except for *T*. *distichum* and *T*. “Zhongshanshan 61”, which have one and three complement repeats, respectively), were identified in the other species (Figure 3A; Supplementary Table S4). In addition, sequence lengths of 30–50 bp were the most common in these cp genomes, especially in *T*. “Zhongshanshan 118” (31, 62%), followed by sequence lengths of >91 bp, then 51–70 bp and 71–90 bp (Figure 3B). The distribution of sequence lengths was even in *T. ascendens*; 12 sequences of 30–50 bp and 17 sequences of greater than 91 bp were identified (Figure 3B; Supplementary Table S4).

Because of the high polymorphism, reproducibility, and abundance of cp SSRs (cpSSRs) in plant genomes, they are some of the most important molecular markers used in genetic analyses of plant populations, evolutionary studies, and breeding programs (Jansen et al., 2007). In our study, mononucleotide and dinucleotide SSRs were the most common cpSSRs in the cp genomes of T. “Zhongshanshan”, and they mainly consisted of A and T. This might be related to the high AT content of the cp genome, which is consistent with the results of studies of the cp genomes of other taxa (Duan et al., 2020; Chen et al., 2022). Repeat sequences are important sources of genomic variation and play key roles in mediating genomic rearrangements; they are commonly used for the development of genetic markers for phylogenetic and population studies (Tangphatsornruang et al., 2010). Of the four long repeat types, forward and palindrome repeats were the most abundant type of repeat in the cp genomes of *Taxodium*, which is consistent with the results of previous studies of the cp genomes of members of the genus *Cupressus* and genus *Juniperus* (Chen et al., 2022). Reverse and complementary repeats were present in *T*. *distichum* and *T. mucronatum* but absent in *T. ascendens*. Furthermore, reverse and complementary repeats were absent in *Cryptomeria duclouxiana*, *J. chinensis*, *J. gaussenii*, *J. pingii*, and *J. procumbens* (Chen et al., 2022). This indicates that there is substantial variation in the frequency of these two repeat types among genera in the family Cupressaceae.

### 3.3 Codon usage and GC content in the cp genomes of *T*. “Zhongshanshan”

**FIGURE 4:**
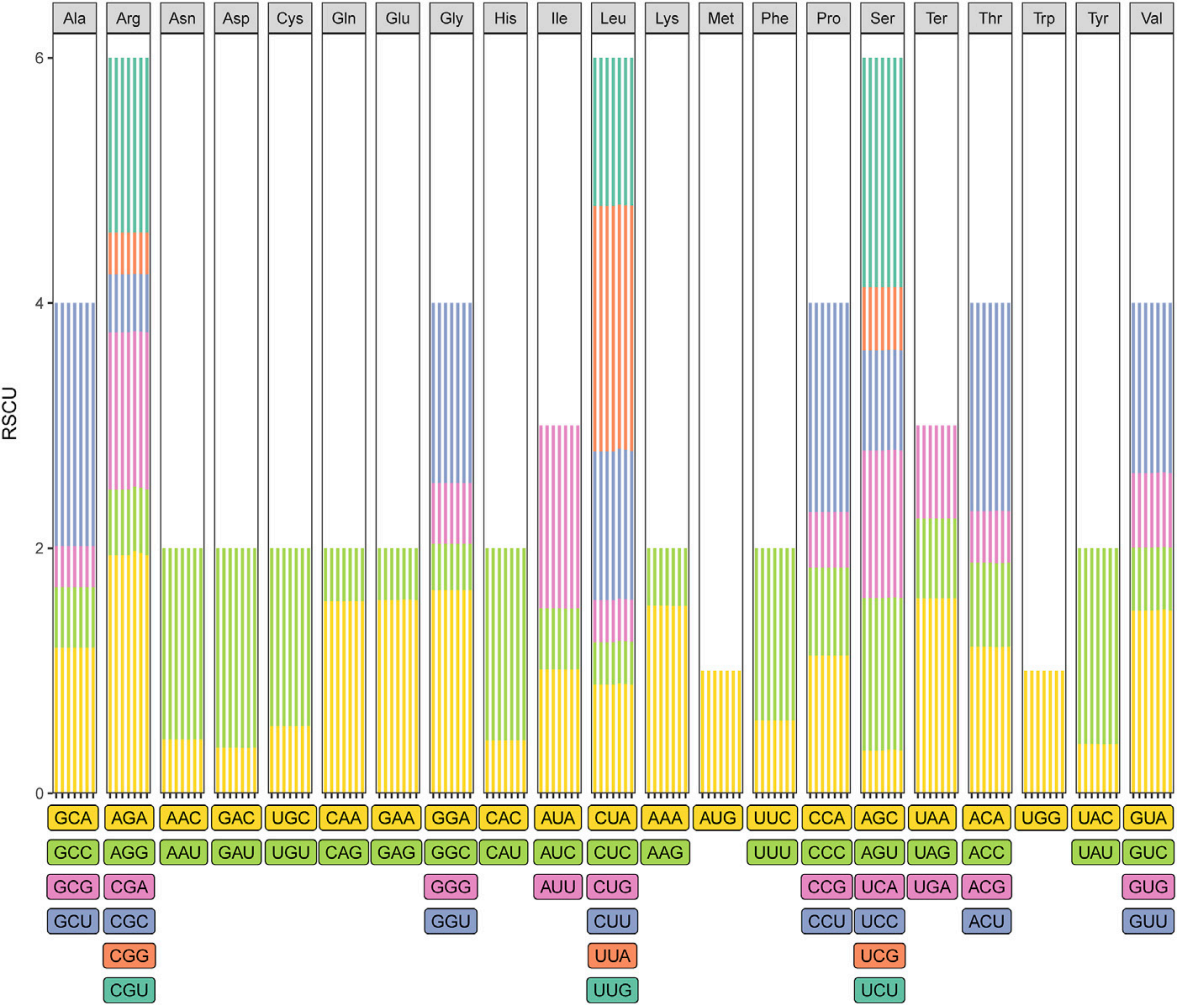
Codon usage in the *Taxodium* cp genomes. Different colors correspond to codons listed underneath the columns. Each histogram from left to right was *T. mucronatum, T*. “Zhongshanshan 401”, *T*. “Zhongshanshan 302”, *T*. “Zhongshanshan 118”, *T. ascendens*, *T.* “Zhongshanshan 61”, and *T. distichum*, respectively.

The usage of synonymous encodes can prevent deleterious mutations, which can result in codon degeneration (Morton, 2003). However, the use of synonymous codons in organisms such as plants is biased (Liu and Xue, 2005), and the relative synonymous codon usage (RSCU) is an indicator of the magnitude of the codon usage bias (Sharp and Li, 1987). We measured the codon usage frequency and RSCU of the seven *Taxodium* species. The total number of codons for protein-coding genes ranged from 24,739 to 24,823, and the patterns of codon usage in *T. mucronatum*, *T*. “Zhongshanshan 118”, *T*. “Zhongshanshan 302”, and *T*. “Zhongshanshan 401” were similar (Figure 4; Supplementary Table S5). Excluding the stop codons, leucine (2,677–2,694 codons, 10.82%–10.85%) was the most abundant amino acid, and cysteine (280 codons, 0.01%) was the least abundant amino acid, respectively, excluding the stop codons. (Figure 4; Supplementary Table S5). Synonymous bias was observed when RSCU A total of 64 codons encoding 21 amino acids were identified. Based on RSCU values, synonymous codon preference can be divided into: no preference (RSCU ≤ 1) and preference (RSCU > 1). In this study, 33 codons showed preference and 31 codons showed no preference for these species. Moreover, except for UUG, most of the preferred codons ended with A or U (Supplementary Table S5).

**FIGURE 5:**
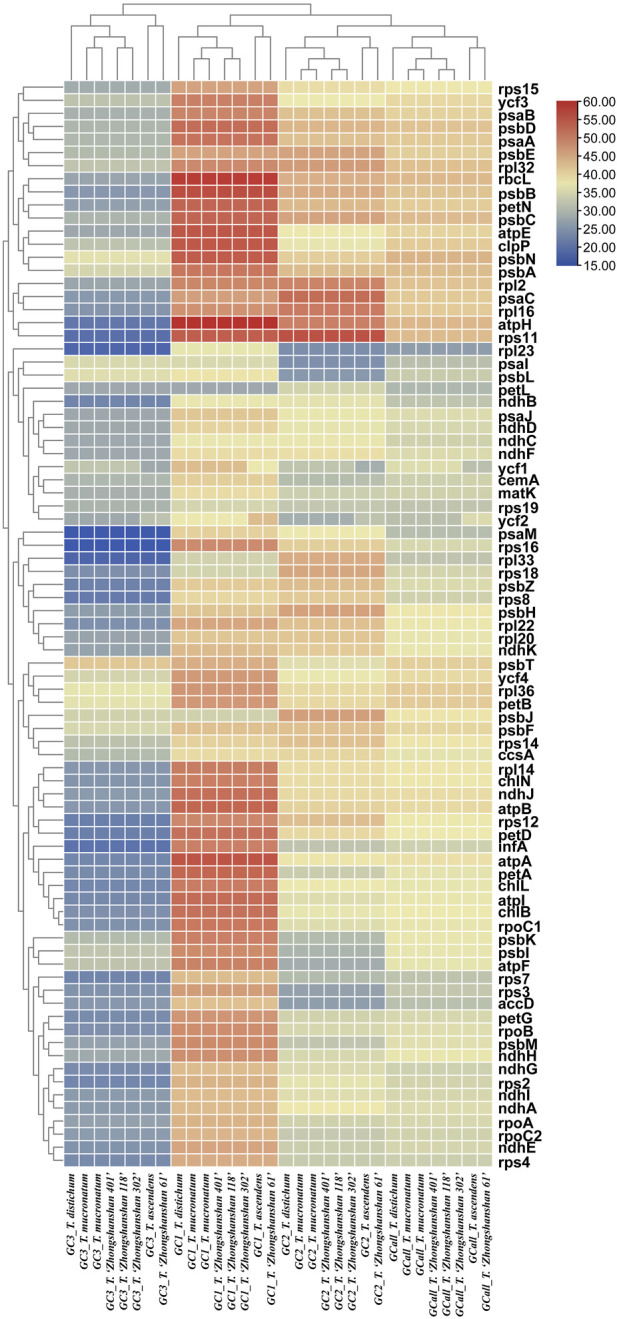
Heat map of the GC content at the first, second, and third positions and the entire codon of all protein-coding genes. The color indicates the percentage of GC content.

The GC content is one of the most important characteristics of cp genomes. Thus, the GC content of the first, second, third positions, and entire codons of 83 protein-coding genes (hereafter referred to as GC1, GC2, GC3, and GCall, respectively) was determined. GC1, GC2, GC3, and GCall showed significant differences between genes but almost no differences between genomes (except for *ycf1* and *ycf2*). Furthermore, the GC content of the *ycf1* and *ycf2* genes (either GC1s, GC2s, GC3s, or GCall) in *T. ascendens* and its offspring *T*. “Zhongshanshan 61” differed significantly from that of other species, indicating that these two genes could be used as markers to distinguish between them. Cluster analysis based on heat maps of GC1s, GC2s, GC3s, and GCall revealed that the four offspring were more closely related to their paternal parents than their maternal parents (Figure 5).

### 3.4 Comparative analysis of the complete cp genomes of *Taxodium* species

**FIGURE 6:**
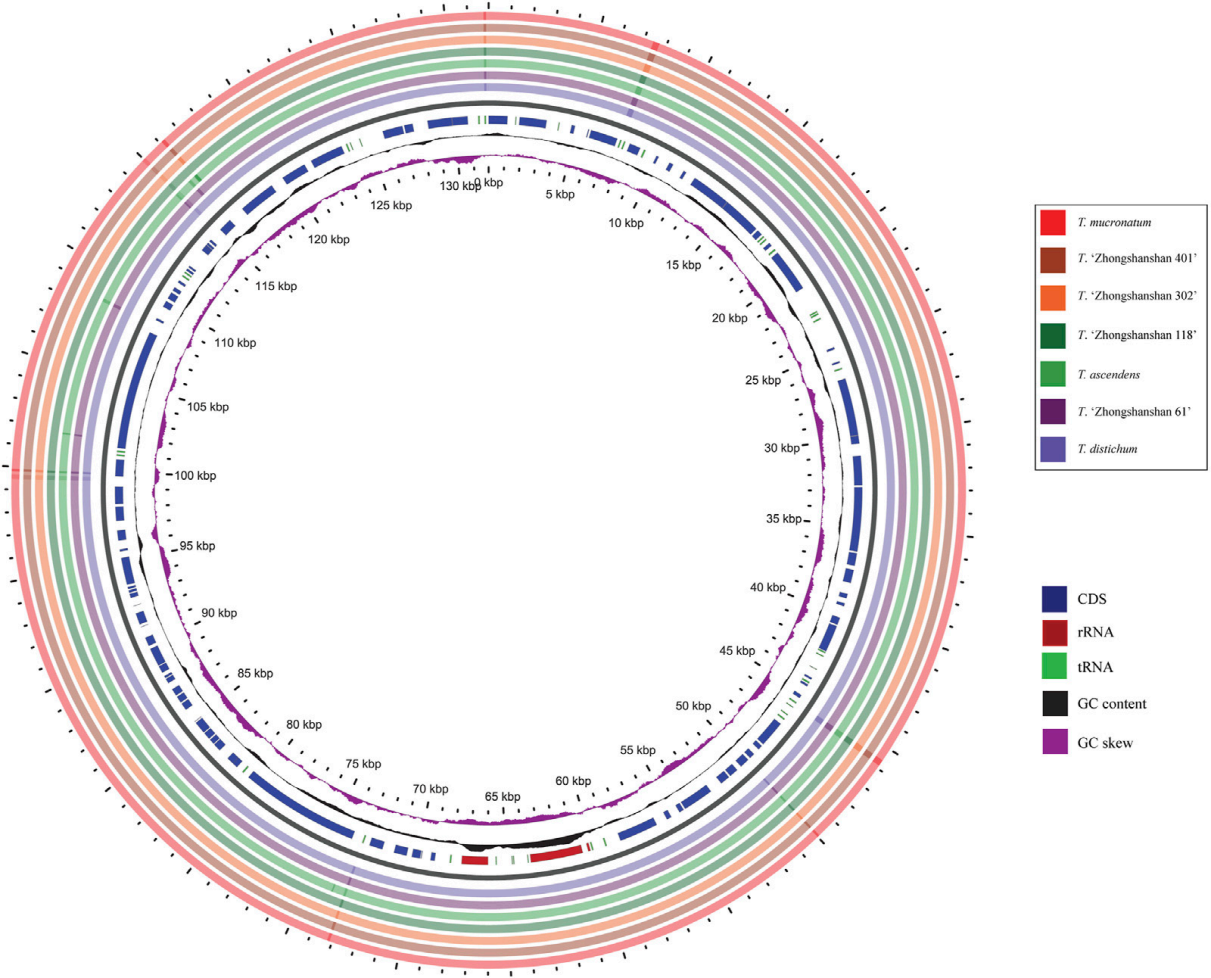
Chloroplast genome comparisons among the seven *Taxodium* species in GView. The seven outermost rings denote the cp genomes sequence comparison by BLAST between *T. distichum* and other species. The innermost purple ring is the GC skew indicating G>C or G < C, the black ring indicates GC content. In the interrupted circles, blue represents coding sequences (CDS), red represents rRNA genes, and green represents tRNA genes.

**FIGURE 7:**
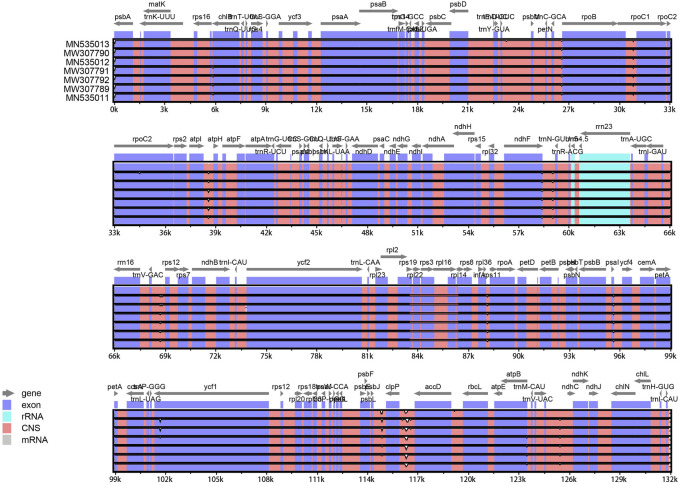
A comparison of the cp genomes among seven *Taxodium* species. The vertical and horizontal axes in the figure represent the percentage of identity ranging from 50% to 100% and the sequence length, respectively. Annotated genes are displayed along the top.

BLAST analysis of the seven *Taxodium* cp genomes was conducted using the mVISTA program and GView software, with *Taxodium distichum* as the reference. The entire cp genome was highly conserved among the examined species, especially the coding regions (Figure 6). Furthermore, divergence in the non-coding regions among species was greater than divergence among coding regions (Figure 7). A high level of divergence was observed for *rps16*-*chlB*, *trnC*-*proB*, *proC1*, *atpI*-*atpH*, *ndhF*-*trnN*, *rrn16*-*trnV*, and *trnV*-*rps12*, which could be suitable candidates for the identification of *Taxodium* species. Additionally, DnaSP software was used to identify hotspots in the cp genomes to characterize patterns of nucleotide variability. Five regions (*trnD*-*psbM*, *atpI*-*atpH*, *psbK*-*trnL*, *trnI*-*rrn16*, and *atpB*-*ndhC*) with *Pi* values were identified (>0.002), and all of these were located in non-coding regions (Supplementary Table S6; Supplementary Figure S1). Additional research is needed to determine whether these regions could be used as molecular markers in phylogenetic studies of *Taxodium* species.

Our findings indicated that the sequences of these seven cp genomes are highly conserved, especially in the coding regions, and the divergence in the non-coding regions among species is greater than the divergence in the coding regions. For example, high variation was observed among the *trnD*-*psbM*, *atpI*-*atpH*, *psbK*-*trnL*, *trnI*-*rrn16*, and *atpB*-*ndhC* regions. Research of these regions will aid future genetic studies as well as the development of molecular markers for the genus *Taxodium*.

### 3.5 Phylogenetic analysis

**FIGURE 8:**
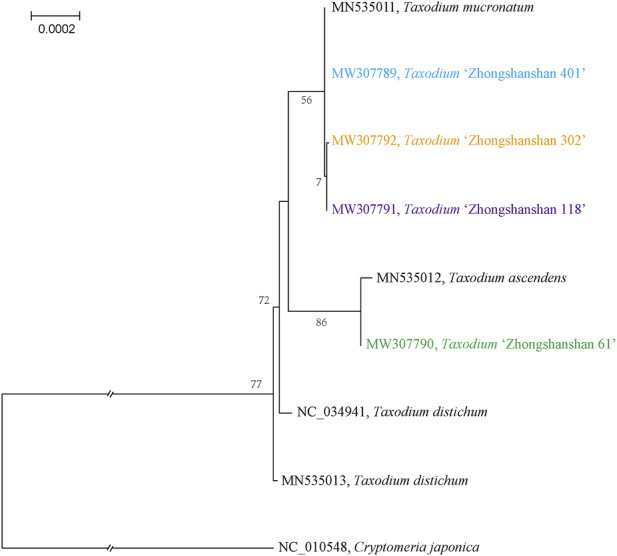
Phylogenetic analysis of *Taxodium* species using their complete cp genomes. Numbers above the nodes are bootstrap values.

To characterize the evolutionary relationships among the four *Taxodium* cultivars obtained, MAFFT was used to align the entire cp genome sequences of the *Taxodium* species. The topology of the phylogenetic tree was visualized using IQ-tree software; the phylogenetic tree was built using the ML method with 1,000 bootstrap replicates and with *Cryptomeria japonica* as the outgroup. *T*. “Zhongshanshan 61” was most closely related to *T. ascendens*, and *T*. “Zhongshanshan 401”, *T*. “Zhongshanshan 302”, *T*. “Zhongshanshan 118”, and *T. mucronatum* were closely related and comprised a subclade (Figure 8).

Two modes of inheritance of cp DNA (cpDNA) have been identified in seed plants cpDNA: uniparental (i.e., maternal or paternal) and biparental (i.e., maternal and paternal) (Hagemann, 1992; Crosby and Smith, 2012). The most common mode of cpDNA inheritance in seed plants is matrilineal, as only a few cases of patrilineal inheritance have been identified to date (Li et al., 2013). In gymnosperms, patrilineal inheritance is the most common mode of inheritance of cpDNA. Ohba (1971) was the first to report the paternal inheritance of cpDNA in reciprocal crosses of *C. japonica* (Ohba, 1971). Other species showing the paternal inheritance of cpDNA since then include *Pinus contora*, *Pinus banksiana* (Dong et al., 1992), *Pinus taeda* (Neale and Sederoff, 1989), and *Sequoia sempervirens* (Neale et al., 1989). The mechanisms underlying the paternal inheritance of cpDNA remain poorly studied. However, a mechanism underlying the paternal inheritance of cpDNA has been identified via studies of the formation of fertilized egg cells in *P. tabulaeformis*. During egg cell formation and development, the cpDNA of the oocyte is eliminated; consequently, no normal plasmid is present in the mature egg cells. At fertilization, the cytoplasm of the parent containing the cpDNA is transferred to the oocyte and then to the proto-embryo. The results of this study provide a cytological mechanism for the paternal inheritance of cpDNA in *Pinus tabulaeformis*. Although the mechanism underlying the paternal inheritance of cpDNA in the genus *Taxodium* remains unclear, the results of this study have implications for genetic studies of *Taxodium* because of the major challenges associated with their large nuclear genomes. For example, the paternal parentage of hybrid offspring in open-pollinated seed orchards can be conveniently identified based on this trait of the cpDNA.

### 3.6 Molecular marker development

**FIGURE 9:**
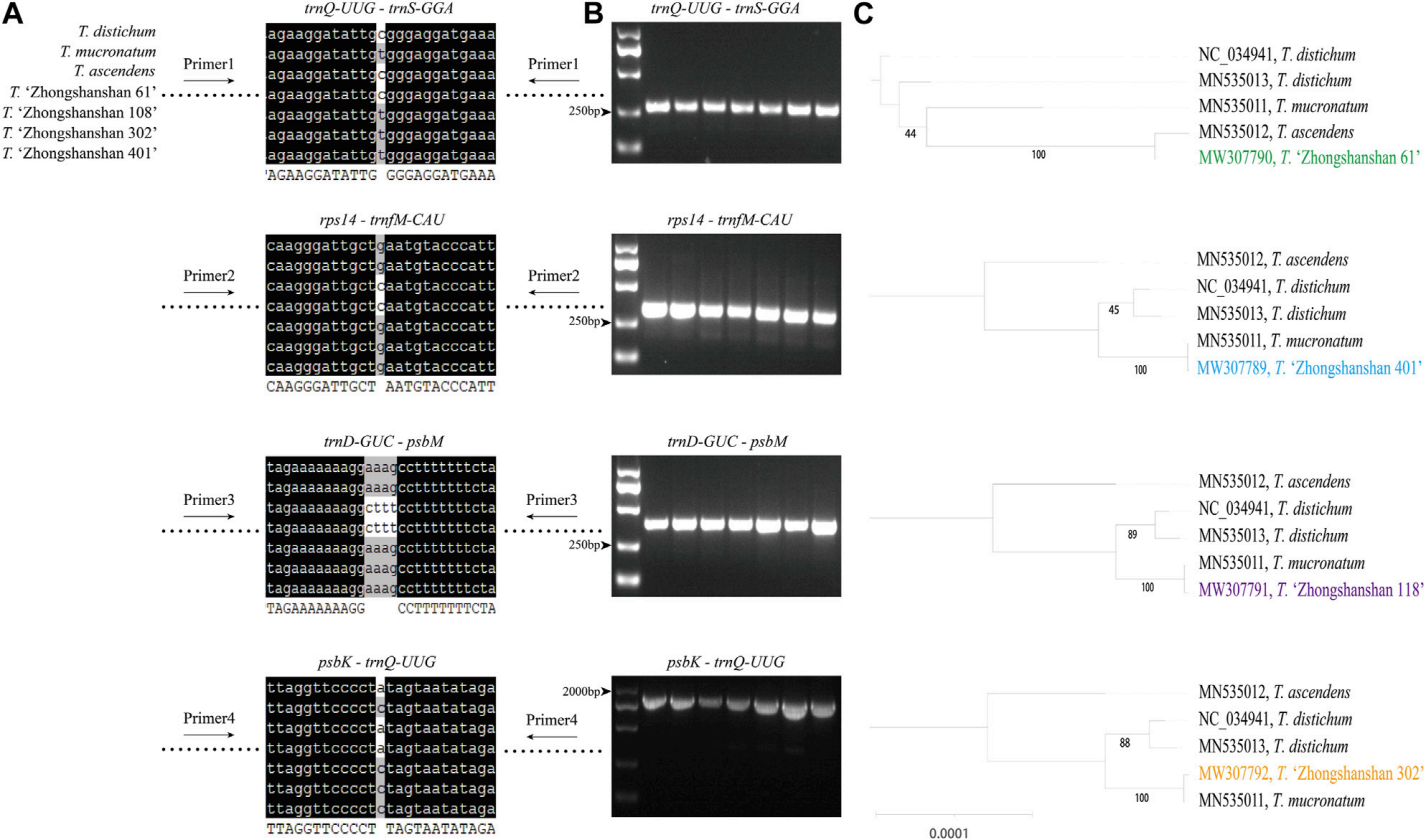
The mutation loci and validation markers for the inheritance of *Taxodium* cp genomes. **(A)** Sequence information of four highly variable regions (blank space means no differences among these sequences); **(B)** Gel electrophoresis results obtained for the four fragments; **(C)** Neighbor-joining tree based on the amplified sequences of the four fragments.

**FIGURE 10:**
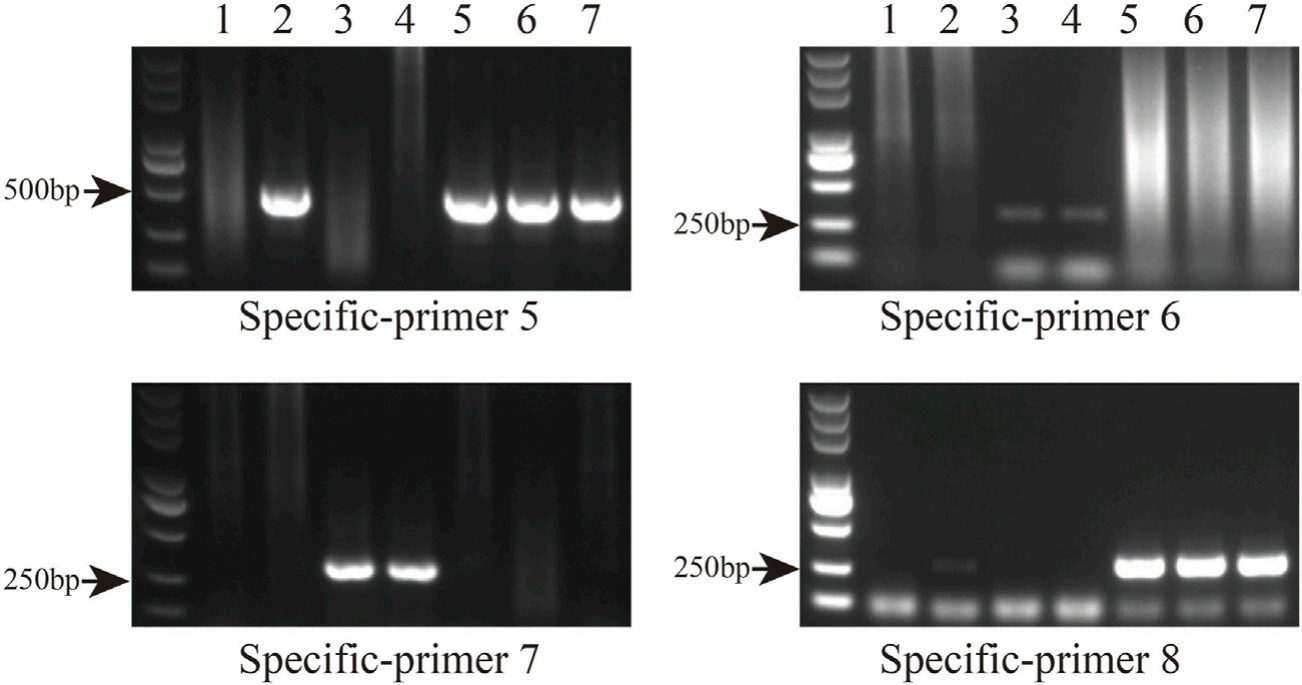
Gel electrophoresis results of specific primers. Amplification results from left to right: *T. distichum*, *T. mucronatum*, *T. ascendens*, *T*. “Zhongshanshan 61,” *T*. “Zhongshanshan 118,” *T*. “Zhongshanshan 302,” and *T*. “Zhongshanshan 401.”

Primer sequences based on polymorphic loci in the cp genomes were designed using four fragments (Figure 9A; Supplementary Table S7). The cp genome of *Taxodium distichum* was used as the reference sequence. First, a neighbor-joining (NJ) tree was built based on the amplified sequences of these four regions (Figure 9B). The results showed that the hybrid offspring clustered with their paternal parents (Figure 9C). In addition, single nucleotide polymorphism (SNP)/insertion and deletion (indel)-based specific primers were developed. Bands were observed for *T*. “Zhongshanshan 118”*, T*. “Zhongshanshan 302”, *T*. “Zhongshanshan 401”, and *T. mucronatum* when specific primers 5 and 8 were used for PCR amplification, and bands for *T. ascendens* and *T*. “Zhongshanshan 61” were only observed when specific primers 6 and 7 were used (Figure 10).

Complete cp genome information can provide insights into plant biology, diversity, and evolutionary relationships (Moore et al., 2007; Shaw et al., 2014). The sequence and structural characteristics of cp genomes make them effective DNA barcodes in plants. Hypervariable regions developed based on the cp genome information are becoming increasingly used for the identification of closely related plant species, such as *Ilex* (Chong et al., 2022), *Cyathula* (Guo et al., 2022), and *Solanum* (Kim and Park, 2019). The results of our study indicated that the cp genome of *Taxodium* is paternally inherited; the specific primers developed in our study could be used for the identification of the male parent of *Taxodium* hybrids. Although verification of these specific markers using other hybrids is needed, these markers could be useful in breeding and genetic research of *Taxodium* spp.

## 4 Conclusion

The complete cp genomes of four *T*. “Zhongshanshan” hybrids were sequenced and compared against the cp genomes of their parents. The full-length cp genomes of these four hybrids ranged from 131,942 to 132,128 bp, and all of the cp genomes contained 120 genes, including 83 protein-coding genes, 33 tRNAs, and four rRNAs. Highly variable regions (e.g., *rps16*-*chlB*, *trnC*-*proB*, *proC1*, *atpI*-*atpH*, *ndhF*-*trnN*, *rrn16*-*trnV*, and *trnV*-*rps12*) were detected, and these regions could aid future genetic and phylogenetic studies. Phylogenetic analysis of the evolutionary relationships based on the GC content of protein-coding genes indicated that the four hybrids were most closely related to their parents. In addition, molecular markers were developed using four hypervariable regions. Neighbor-joining trees were constructed based on PCR-amplified regions, as well as gel electrophoresis of specific primers, which were developed based on the SNPs/indels in these regions. Our findings suggest that these markers could be used to identify the male parent of the *Taxodium* hybrids. To our knowledge, this is the first study to demonstrate that the cp genomes of *Taxodium* are paternally inherited. The molecular markers developed in our study will aid *Taxodium* breeding research*.*

## Data Availability

The datasets presented in this study can be found in online repositories. The names of the repository/repositories and accession number(s) can be found below: https://www.ncbi.nlm.nih.gov/genbank/, MW307790 https://www.ncbi.nlm.nih.gov/genbank/, MW307791 https://www.ncbi.nlm.nih.gov/genbank/, MW307792 https://www.ncbi.nlm.nih.gov/, SRR13347061 https://www.ncbi.nlm.nih.gov/, SRR13347062 https://www.ncbi.nlm.nih.gov/, SRR13347063 https://www.ncbi.nlm.nih.gov/, SRR13347064.
