# Supplementary figures and images for: Novel molecular markers for Taxodium breeding from the chloroplast genomes of four artificial Taxodium hybrids
Source: Front Genet. 2023 Aug 2;14:1193023. doi: 10.3389/fgene.2023.1193023 (PMC10433758; doi:10.3389/fgene.2023.1193023)

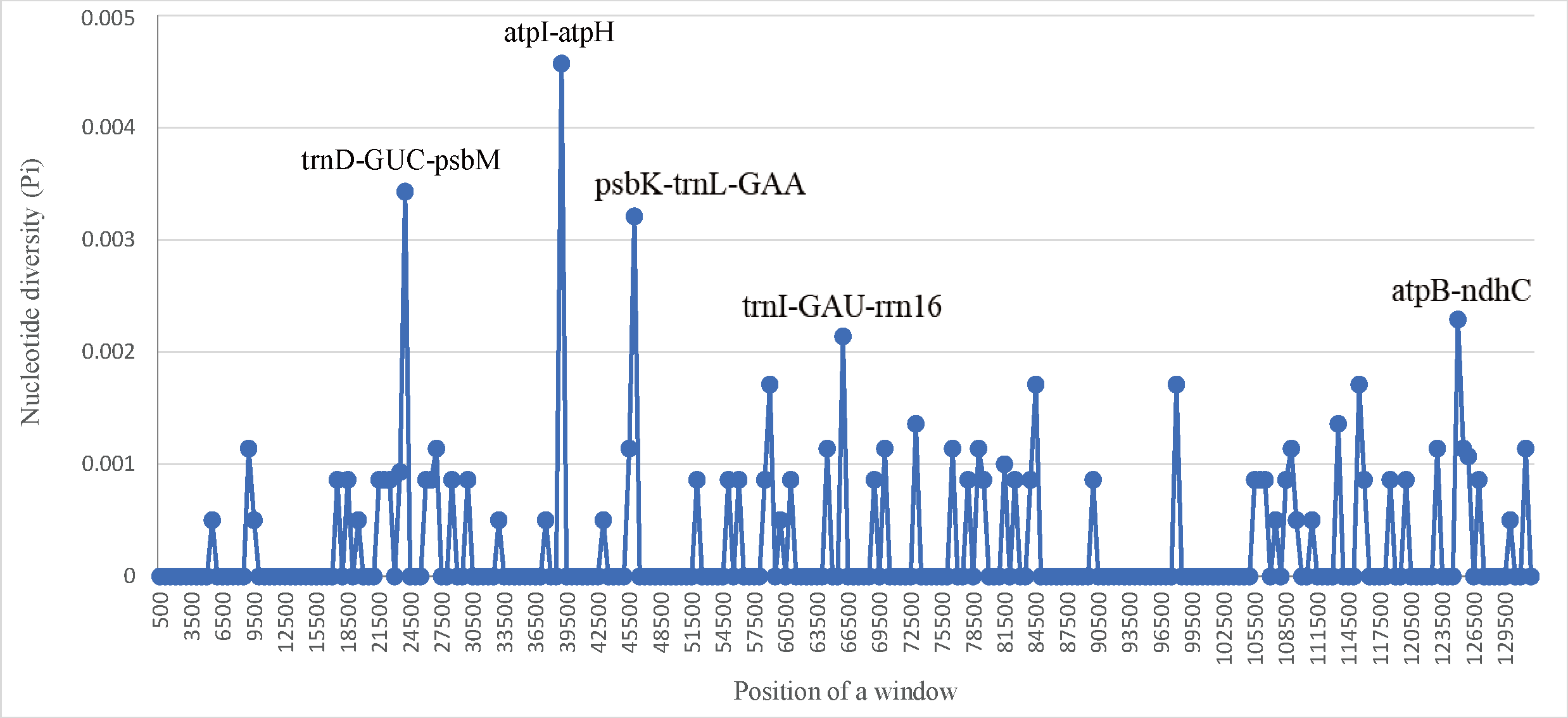

Supplement: Supplementary file 6 [file Image1.PNG]
